# Chinese healthy eating index as a measure of diet quality and its association with depression and anxiety symptoms among people living with HIV: a cross-sectional study in China

**DOI:** 10.3389/fnut.2026.1748219

**Published:** 2026-02-04

**Authors:** Huiling Tang, Zizheng Nie, Chenyang Wu, Junxia Cheng, Ke Zhu, Yingying Liu, Mu Zhang, Fengying Wang, Shufen Han

**Affiliations:** 1 Jinhua Center for Disease Prevention and Control, Jinhua, Zhejiang, China; 2 School of Public Health and Nursing, Hangzhou Normal University, Hangzhou, Zhejiang, China

**Keywords:** Chinese healthy eating index, depression, anxiety, HIV, cross-sectional study

## Abstract

**Aim:**

High diet quality has increasingly been acknowledged as a significant factor influencing mental health. This study aimed to investigate the association between the Chinese Healthy Eating Index (CHEI) and mental health issues, specifically depression and anxiety, among people living with human immunodeficiency (PLHIV).

**Design and methods:**

In the cross-sectional study, data were collected from 700 PLHIV through in-person interviews conducted at HIV-specific hospitals in Jinhua city. Dietary intake and food information were obtained using a semi-quantitative food frequency questionnaire, and diet quality was assessed through the CHEI. Depression and anxiety were evaluated using the Hospital Anxiety and Depression Scale (HADS). Multivariate logistic regression models and restricted cubic spline (RCS) analysis were used to examine their associations.

**Results:**

The average total CHEI scores were 60.8 ± 12.0. After adjusting for potential confounders, participants in the highest quartile of total CHEI scores exhibited a significantly lower likelihood of experiencing symptoms of depression (odds ratio [OR] = 0.35; 95% confidence interval [CI]: 0.20–0.60) and anxiety (OR = 0.57; 95% CI: 0.34–0.96) compared to those in the lowest quartile. The RCS analysis indicated that as total CHEI scores exceeded 60, the risk of experiencing depression and anxiety symptoms showed a declining trend. Furthermore, higher CHEI’s component scores for dark vegetables, fruits, and dairy products were associated with lower odds of experiencing depression and anxiety symptoms within this population.

**Conclusion:**

Our findings indicated that diet quality, as evaluated by the CHEI, was inversely associated with symptoms of depression and anxiety among PLHIV.

## 1 Introduction

Mental disorders are among the leading contributors to the global public health burden (1), with an estimated 970 million people affected worldwide in 2009. People living with human immunodeficiency virus (PLHIV) are more susceptible to experiencing severe psychological changes and vulnerabilities associated with mood disturbances. Depression and anxiety are among the most common mental disorders in this population, substantially impacting their quality of life and contributing to a reduced life expectancy (2). Estimates of depression prevalence among PLHIV vary significantly, ranging from 22% in Europe to 44% in South America (3). This starkly contrasts with the approximate prevalence rates of 5% in the general adult population and 5.7% in individuals aged 60 or older (4). Additionally, the average prevalence of anxiety within this population is approximately 15.5% (5), which adversely affects HIV prognosis, prolongs the time required to achieve viral suppression, and increases the likelihood of antiretroviral treatment failure even after suppression (6). Implementing an integrated approach that simultaneously addresses both depression and anxiety is crucial for improving outcomes for PLHIV (7, 8). Recently, there has been a growing emphasis on the management of these mental health conditions through improvements in diet quality (9, 10). Population studies have shown that a better quality diet is associated with better mental health outcomes (10–13). For PLHIV, poor diet quality, characterized by a low intake of fruits and vegetables, and a high consumption of saturated fats and processed carbohydrates, may exacerbate disease progression and increase the risk of depression and anxiety through promoting inflammatory responses and disrupting the balance of the gut microbiome (14–16).

The Healthy Eating Index (HEI) was first established in 1995 to evaluate diet quality based on the consumption of foods recommended by the Dietary Guidelines for Americans (DGA) (17), and it has undergone several revisions in conjunction with updates to the DGA, including the DGA-2005, DGA-2010, DGA-2015, and DGA-2020. This index functions as a continuous scoring system used to evaluate diet quality and to analyze the associations between dietary habits and specific health outcomes. Following the updated Dietary Guidelines for Chinese Residents 2016 (DGC-2016), the Chinese Healthy Eating Index (CHEI) was developed based on the HEI framework (18), which has been utilized to investigate the relationship between dietary patterns and various disorders, including metabolic syndrome (19, 20) and diabetes (21), among Chinese adults. Several recent studies have indicated that a higher HEI-2015 score was associated with a reduced likelihood of experiencing depression and anxiety symptoms (22, 23). However, the CHEI, reflecting traditional Chinese dietary patterns, has not been studied in this context, particularly among PLHIV, who may experience distinct dietary habits and nutritional challenges. Consequently, we hypothesized that PLHIV who have higher CHEI scores would exhibit lower odds of experiencing depression and anxiety symptoms. To test this hypothesis, we applied an updated version of the CHEI to assess diet quality in this population. We examined its association with mental health outcomes through a cross-sectional study conducted in China.

## 2 Methods

### 2.1 Study design and participants

This cross-sectional study was designed to examine the CHEI as a measure of diet quality and its relationship with the mental health of PLHIV in Jinhua City, Zhejiang Province. The design and implementation of this cross-sectional study were rigorously in accordance with the STROBE guidelines. The study was conducted between December 2023 and June 2024. The present study adhered to the ethical principles outlined in the World Medical Association’s Declaration of Helsinki and the Good Clinical Practice guidelines for medical research involving human subjects. Approval was obtained from the Ethics Committee of the Jinhua Center for Disease Prevention and Control (No. 2024–2) and the Institutional Review Board of Medical Research, School of Public Health, Hangzhou Normal University (No. 202300010). All protocols were performed in accordance with the Declaration of Helsinki.

All PLHIV receive their care at local Centers for Disease Control and Prevention (CDC). The study used a convenience sampling method to recruit PLHIV who were receiving regular checkups at the HIV outpatient clinic of designated hospitals. The inclusion criteria were as follows: participants aged 18 years and older who were receiving antiretroviral therapy following their diagnosis, individuals demonstrating adequate language expression and comprehension, and those who provided informed consent for study participation. Informed consent was obtained from all participants who voluntarily agreed to participate in this study. The exclusion criteria were as follows: HIV-positive pregnant or breastfeeding women, individuals with severe physical disabilities or cognitive impairments, those with a history of psychiatric disorders prior to their HIV diagnosis, and individuals who had previously participated in stress protocols.

### 2.2 Estimation of sample size

Based on relevant studies, the expected prevalence among PLHIV was estimated at approximately 31.0% (3). With a permissible error of 0.04 and a $Z_{1-\alpha /2}$ value of 1.96, we calculated the required sample size using the corresponding formula $n=(Z_{1-\alpha /2}^{2}\times p\times q)/d^{2}$, which yielded an initial estimate of 506 participants. After accounting for an anticipated 20% loss to follow-up, the final sample size was set at no fewer than 600 participants.

### 2.3 Data collection and detection of biochemical parameters

Participants included in the study were recruited for in-person interviews by trained medical graduate students using a validated written questionnaire. PLHIV who met the eligibility criteria were informed about the study’s objectives and benefits, as well as the principles of confidentiality and the measures implemented to safeguard their personal information. The general information included sociodemographic details, such as age, sex, region of residence, education level, floating population status, personal monthly income, marital status, and body mass index (BMI). The term “floating population” refers to individuals who reside in a specific locality for less than 6 months. Furthermore, we documented lifestyle habits, including smoking status, physical activity, and sedentary behavior, as well as infection-related factors such as routes of infection, duration of disease and complications, cluster of differentiation 4-positive (CD4^+^) T lymphocyte counts, medication side effects, and dietary intake. Data on physical activity were collected using a shortened version of the International Physical Activity Questionnaire (IPAQ). Based on the established classification standards (24), the responses were subsequently classified into three levels: low, moderate, and high. Data on infection-related factors were extracted from the databases of the HIV outpatient clinic at designated hospitals and the CDC in Jinhua city. Recent counts of CD4^+^ T lymphocytes in blood samples were detected using flow cytometry. According to the diagnostic criteria for acquired immunodeficiency syndrome (AIDS) and HIV infection established in China, individuals infected with HIV progress through three CD4 stages prior to being diagnosed with AIDS, which is defined by CD4^+^ cell counts of less than 200/mm^3^. The three stages are as follows: no immune deficiency (CD4^+^ cell counts ≥ 500/mm^3^), mild immune deficiency (CD4^+^ cell counts ranging from 350/mm^3^ to 499/mm^3^), and moderate immune deficiency (CD4^+^ cell counts between 200/mm^3^ and 349/mm^3^).

### 2.4 Dietary questionnaire

Dietary data were collected by trained medical graduate students using a semi-quantitative food frequency questionnaire (FFQ) previously validated in the Chinese population (25). Face-to-face interviews and food models were employed to improve the accuracy of reported dietary intake collected from PLHIV over the past month. First, participants reported whether they had consumed each specified food item in the past month. Subsequently, they reported their frequency of consumption by selecting one of the following options: (1) never consumed; (2) occurrences per month; (3) occurrences per week; or (4) occurrences per day. The actual quantities of food consumed for each item were estimated as daily intake amounts and used for subsequent analyses. The Cronbach’s *α* coefficient for this dietary survey was 0.64, indicating acceptable internal consistency.

### 2.5 Calculation of CHEI

The total CHEI score, used to assess diet quality, was calculated according to the methodologies established in the existing literature (18, 19). Briefly, the CHEI—a continuous scoring system—comprises 17 food components, including 12 recommended food groups for adequate intake and 5 categories designated for limited intake. The updated version of the CHEI represents a refined adaptation of the CHEI-2016 and aligns with the DGC-2022 food recommendations (19). Standard proportions are used to uniformly quantify different types of food. Fruits, cooking oils, and sodium were assigned scores ranging from 0 to 10, while all other components were scored between 0 and 5. Each component’s score was calculated and then aggregated across all 17 components to derive a total CHEI score, ranging from 0 to 100. The total CHEI scores are categorized into quartiles (Q) according to specific cutoff points: Q1 (25.7–52.4), Q2 (52.5–60.8), Q3 (60.9–69.8), and Q4 (69.9–97.6). Higher total CHEI scores indicate better diet quality and greater adherence to the DGC-2022 guidelines.

### 2.6 Assessment for depression and anxiety symptoms

Participants’ symptoms of depression and anxiety were evaluated using the Hospital Anxiety and Depression Scale (HADS) (26), which comprises 14 items divided into two subscales: the Depression Subscale (HADS-D) and the Anxiety Subscale (HADS-A). Each subscale yields scores ranging from 0 to 21. The subscales were designed to differentiate between depression and anxiety symptoms, while minimizing the influence of somatic symptoms or other severe mental disorders (27). A cut-off score of eight or higher on each subscale indicates an optimal balance between sensitivity and specificity for identifying symptoms of depression and anxiety disorders (26). The Cronbach’s *α* coefficients were 0.89 for the Depression Subscale and 0.90 for the Anxiety Subscale, demonstrating good internal consistency for both subscales (average Cronbach’s α = 0.82 for HADS-D and 0.83 for HADS-A).

### 2.7 Statistical analyses

All participants were grouped into four quartiles based on their total CHEI scores. For baseline characteristics, quantitative data were described as medians with interquartile ranges and examined using the Mann–Whitney test or the Kruskal–Wallis test, while categorical data were expressed as frequencies and percentages (%), and analyzed using the chi-squared test or Fisher’s exact test. A univariable binary logistic regression was conducted to investigate the association between total CHEI scores and the risk of depression and anxiety symptoms among Chinese PLHIV. To calculate odds ratios (ORs) and their 95% confidence intervals (CIs), the lowest total CHEI scores—specifically the first quartile—were designated as the reference category. Model 1 was adjusted for age, sex, education level, floating population status, monthly income, and marital status, based on the crude model. Model 2 was additionally adjusted for BMI, smoking status, physical activity level, and sedentary behavior. Model 3 was further adjusted for duration of infection, CD4^+^ T lymphocyte counts, complications, and medication side effects. Restricted cubic spline (RCS) analysis with three knots at the 10th, 50th, and 90th percentiles of CHEI was used to explore whether a dose–response linear relationship existed between total CHEI scores and the risk of depression and anxiety. These specific percentiles were selected to effectively represent the data, approximately corresponding to low, median, and high CHEI values. This selection is a conventional method for modeling non-linear trends while mitigating the risk of overfitting.

To evaluate potential effect modifications, we further performed subgroup analyses based on age (≥45 years and <45 years), sex (male and female), education level (primary school or below, junior middle school, senior high school or vocational school, and junior college or above), floating population (no and yes), monthly income (≤CNY ¥ 3,000, CNY ¥3,001–5,000, CNY ¥5,001–8,000, and > CNY ¥8,000), marital status (married, unmarried, and divorce or widowed), BMI (≥24 kg/m^2^ and <24 kg/m^2^), smoking status (no and yes), physical activity (low level, medium level, and high level), sedentary behavior (no and yes), duration of infection (≥6 years and <6 years), CD4^+^ T lymphocyte counts (<350/mm^3^, 350–499/mm^3^ and >500/mm^3^), complications (no and yes) and medication side effects (no and yes). For each stratification factor, we included a multiplicative interaction term between total CHEI scores and the subgroups in the multivariate models to calculate *p*-values for interaction (*p*-interaction).

All statistical analyses were performed using R software (version 4.3.3, R Development Core Team, Vienna, Austria). A *p*-value of less than 0.05 was considered statistically significant.

## 3 Results

### 3.1 Baseline characteristics of participants

A total of 762 HIV-positive individuals participated in this survey. Based on the questionnaire’s integrity and rationality, we excluded responses containing incorrect information, resulting in a final analysis including 700 PLHIV (588 men and 112 women). The median age of the participants was 38.0 (IQR: 30.0, 49.0) years, and the median duration of infection was 6.0 (IQR: 3.0, 9.0) years. Table 1 presents the general characteristics of the enrolled PLHIV, categorized by quartiles of their total CHEI scores. PLHIV who had higher total CHEI scores were more likely to be female, part of the settled population, and non-smokers, and they exhibited higher levels of physical activity along with lower levels of sedentary behavior. Furthermore, this group reported higher intakes of whole grains and mixed beans, tubers, dark vegetables, fruits, dairy products, soybeans, fish and seafood, poultry, eggs, and nuts, while consuming lower amounts of red meat.

**Table 1:** General characteristics of participants according to the Chinese healthy eating index.

Characteristics	Total (*n* = 700)	Quartiles of the average CHEI score				*p*-value
		Q1 (*n* = 175)	Q2 (*n* = 175)	Q3 (*n* = 175)	Q4 (*n* = 175)	
Age, years	38.0 (30.0, 49.0)	37.0 (29.0, 47.5)	36.00 (29.0, 47.0)	39.0 (32.0, 51.0)	39.0 (31.0, 48.0)	0.055
Male, n (%)	588 (84.0)	155 (88.6)	154 (88.0)	147 (84.0)	132 (75.4)	<0.001
Urban residence, n (%)	404 (57.7)	107 (61.1)	95 (54.3)	96 (54.9)	106 (60.6)	0.946
Education level, n (%)						0.701
Primary school or below	115 (16.4)	28 (16.0)	25 (14.3)	31 (17.7)	31 (17.7)	
Junior middle school	168 (24.0)	44 (25.1)	47 (26.9)	39 (22.3)	38 (21.7)	
Senior high school or vocational school	149 (21.3)	36 (20.6)	42 (24.0)	34 (19.4)	37 (21.2)	
Junior college or above	268 (38.3)	67 (38.3)	61 (34.8)	71 (40.6)	69 (39.4)	
Floating population, n (%)						0.013
No	341 (48.7)	73 (41.7)	79 (45.1)	98 (56.0)	91 (52.0)	
Yes	359 (51.3)	102 (58.3)	96 (54.9)	77 (44.0)	84 (48.0)	
Monthly income, n(%)						0.190
≤CNY ¥ 3,000	141 (20.1)	25 (14.3)	41 (23.4)	39 (22.3)	36 (20.6)	
CNY ¥ 3,001–5,000	216 (30.9)	57 (32.6)	59 (33.7)	57 (32.6)	43 (24.6)	
CNY ¥ 5,001–8,000	209 (29.9)	60 (34.3)	49 (28.0)	44 (25.1)	56 (32.0)	
> CNY ¥ 8,000	134 (19.1)	33 (18.8)	26 (14.9)	35 (20.0)	40 (22.8)	
Marital status, n (%)						0.187
Married	366 (52.3)	49 (28.0)	51 (29.1)	62 (35.4)	62 (35.4)	
Unmarried	224 (32.0)	99 (56.6)	98 (56.0)	78 (44.6)	91 (52.0)	
Divorce or widowhood	110 (15.7)	27 (15.4)	26 (14.9)	35 (20.0)	22 (12.6)	
BMI, n (%)						0.733
<18.5 kg/m^2^	56 (8.0)	13 (7.4)	16 (9.2)	15 (8.6)	12 (6.9)	
18.5–24.0 kg/m^2^	420 (60.0)	105 (60.0)	102 (58.3)	103 (58.9)	110 (62.9)	
24.0–-28.0 kg/m^2^	183 (26.1)	43 (24.6)	48 (27.4)	48 (27.4)	44 (25.1)	
≥28.0 kg/m^2^	41 (5.9)	14 (8.0)	9 (5.1)	9 (5.1)	9 (5.1)	
Smoking status, n (%)						<0.001
No	498 (71.1)	99 (56.6)	123 (70.3)	136 (77.7)	140 (80.0)	
Yes	202 (28.9)	76 (43.4)	52 (29.7)	39 (22.3)	35 (20.0)	
Physical activity, n (%)						<0.001
Low level	417 (59.6)	128 (73.2)	110 (62.9)	96 (54.9)	83 (47.4)	
Medium level	181 (25.8)	30 (17.1)	41 (23.4)	51 (29.1)	59 (33.7)	
High level	102 (14.6)	17 (9.7)	24 (13.7)	28 (16.0)	33 (18.9)	
Sedentary behavior, n (%)						0.001
Yes	506 (72.3)	112 (64.0)	122 (69.7)	136 (77.7)	136 (77.7)	
No	194 (27.7)	63 (36.0)	53 (30.3)	39 (22.3)	39 (22.3)	
Routes of infection, n (%)						0.859
Heterosexual transmission	363 (51.9)	94 (53.7)	88 (50.3)	86 (49.2)	95 (54.3)	
Homosexual transmission	320 (45.7)	77 (44.0)	82 (46.9)	83 (47.4)	78 (44.6)	
Others	17 (2.4)	4 (2.3)	5 (2.8)	6 (3.4)	2 (1.1)	
Duration of infection, years	6.0 (3.0, 9.0)	5.0 (2.0, 8.0)	6.0 (2.0, 8.0)	6.0 (3.0, 9.0)	6.0 (3.0, 9.0)	0.078
CD4^+^ T lymphocytes, n (%)						0.394
<350 /mm^3^	120 (17.9)	23 (13.86)	34 (20.86)	34 (20.00)	29 (16.96)	
350–499 /mm^3^	224 (33.4)	66 (39.76)	50 (30.67)	53 (31.18)	55 (32.16)	
>500 /mm^3^	326 (48.7)	77 (46.39)	79 (48.47)	83 (48.82)	87 (50.88)	
Complications, n (%)						0.249
No		119 (68.0)	123 (70.3)	127 (72.6)	128 (73.1)	
Yes		56 (32.0)	52 (29.7)	48 (27.4)	47 (26.9)	
Medication side effects, n (%)						0.327
No	497	129 (73.7)	126 (72.0)	133 (76.0)	118 (67.4)	
Yes	203	46 (26.3)	49 (28.0)	42 (24.0)	57 (32.6)	
Cumulative average dietary intake						
Total grains, g/day	254.2 (200.0, 360.8)	290.0 (204.2, 403.3)	256.7(200.0, 376.7)	250.0 (198.3, 326.7)	240.0 (166.7, 345.8)	0.043
Whole grains and mixed beans, g/day	10.0 (0.0, 20.0)	0.0 (0.0, 10.0)	6.7 (0.0, 10.0)	10.0 (0.0, 20.0)	20.0 (10.0, 50.0)	<0.001
Tubers, g/day	10.0 (0.0, 33.3)	5.0 (0.0, 13.3)	10.00 (0.0, 20.0)	16.7 (9.2, 45.0)	26.7 (10.0, 51.7)	<0.001
Total vegetables, g/day	153.7 (83.0, 240.0)	103.3 (40.0, 200.0)	133.3 (70.0, 220.0)	173.3 (100.0, 233.3)	216.7 (133.3, 366.7)	<0.001
Dark vegetables, g/day	66.7 (33.3, 100.0)	33.3 (13.3, 95.0)	66.7 (20.0, 100.0)	66.67 (33.3, 100.0)	100.00 (66.7, 150.0)	<0.001
Fruits, g/day	100.0 (33.3, 200.0)	33.3 (13.3, 91.7)	66.7 (26.7, 200.0)	133.3 (66.7, 200.0)	200.0 (133.3, 200.0)	<0.001
Dairy, g/day	66.7 (13.3, 180.0)	30.0 (0.0, 83.3)	33.3 (0.0, 166.7)	83.3 (21.7, 226.7)	150.0 (60.0, 250.0)	<0.001
Soybeans, g/day	7.9 (2.6, 13.5)	3.5 (1.7, 10.4)	6.1 (1.7, 13.1)	8.6 (3.4, 13.5)	12.3 (4.9, 18.4)	<0.001
Fish and seafood, g/day	16.7 (6.7, 37.3)	8.0 (3.3, 20.0)	15.3 (6.7, 33.3)	16.7 (6.7, 33.3)	33.3 (10.7, 66.7)	<0.001
Poultry, g/day	16.7 (3.3, 40.0)	6.7 (0.0, 30.0)	16.7 (3.3, 33.3)	16.7 (2.7, 33.3)	23.3 (6.7, 50.0)	<0.001
Eggs, g/day	38.7 (16.7, 58.7)	18.7 (6.7, 50.0)	33.3 (16.7, 50.5)	43.3 (17.3, 65.7)	50.0 (33.3, 80.0)	<0.001
Nuts, g/day	2.0 (0.0, 8.33)	1.3 (0.0, 3.3)	1.7 (0.0, 3.3)	3.33 (1.3, 8.3)	8.3 (2.3, 11.7)	<0.001
Red meat, g/day	66.7 (33.3, 133.3)	100.0 (43.3, 200.0)	75.0 (50.0, 150.0)	66.7 (33.3, 101.7)	56.7 (33.3, 100.0)	<0.001
Added sugars, g/day	8.0 (0.0, 26.7)	7.3 (0.0, 21.5)	8.0 (0.0, 24.0)	7.0 (0.0, 23.8)	11.0 (0.0, 33.2)	0.206
Alcohol, g/day	0.0 (0.0, 0.0)	0.0 (0.0, 16.0)	0.0 (0.0, 7.6)	0.0 (0.0, 0.0)	0.0 (0.0, 0.0)	<0.001

Categorical variables are presented as numbers and percentages (%), and continuous variables are presented as median and interquartile range. *p*-values were calculated using the Mann–Whitney test or the Kruskal–Wallis test for continuous variables, and the chi-squared test or Fisher’s exact test for categorical variables. BMI, body mass index; Q, quartiles of average CHEI score.

### 3.2 Total CHEI scores and their association with depression and anxiety symptoms

The total CHEI scores for PLHIV ranged from 25.7 to 97.6, with an average score of 60.8 ± 12.0. PLHIV who experienced symptoms of depression (57.7 ± 11.2 vs. 62.1 ± 12.1) or anxiety (58.7 ± 12.0 vs. 61.8 ± 11.9) had lower total CHEI scores compared to those without these symptoms. Table 2 shows the crude and multivariable-adjusted ORs along with their corresponding 95% CIs for depression and anxiety symptoms, categorized by quartiles of their total CHEI scores. After adjusting for potential confounders, PLHIV in the highest quartile of total CHEI scores had significantly lower odds of experiencing symptoms of depression (OR = 0.35; 95% CI: 0.20–0.60) and anxiety (OR = 0.57; 95% CI: 0.34–0.96) compared to those in the lowest quartile. Furthermore, a one-standard-deviation increase in total CHEI scores (12.0 points) was associated with a 33% reduction in the risk of depression (OR = 0.67; 95% CI: 0.55–0.81) and a 23% reduction in the risk of anxiety (OR = 0.77; 95% CI: 0.64–0.93). Additionally, a 5-point increase in total CHEI scores was associated with a 15% lower risk of depression (OR = 0.85; 95% CI: 0.78–0.92) and a 10% lower risk of anxiety (OR = 0.90; 95% CI: 0.84–0.97).

**Table 2:** Association of average CHEI scores with symptoms of depression and anxiety among Chinese people living with HIV.

CHEI	OR (95% CI)				Per SD (12.0) increase	Per 5-point increase	*p*-trend
	Q1	Q2	Q3	Q4			
Total (*n* = 700)	175	175	175	175	700	700	
Score range	25.7–52.4	52.4–60.8	60.8–69.8	69.8–97.6	25.7–97.6	25.7–97.6	
Median	46.6	56.4	65.2	74.9	60.8	60.8	
CHEI scores and depression symptoms							
Crude Model	Ref.	0.84 (0.54, 1.30)	0.51 (0.32, 0.80)	0.40 (0.25, 0.65)	0.69 (0.58, 0.81)	0.86 (0.80, 0.92)	<0.001
Model 1	Ref.	0.77 (0.49, 1.22)	0.48 (0.29, 0.77)	0.36 (0.21, 0.59)	0.66 (0.56, 0.79)	0.84 (0.78, 0.91)	<0.001
Model 2	Ref.	0.80 (0.50, 1.28)	0.52 (0.32, 0.87)	0.41 (0.24, 0.69)	0.69 (0.57, 0.83)	0.86 (0.79, 0.93)	<0.001
Model 3	Ref.	0.77 (0.47, 1.24)	0.49 (0.29, 0.83)	0.35 (0.20, 0.60)	0.67 (0.55, 0.81)	0.85 (0.78, 0.92)	<0.001
CHEI scores and anxiety symptoms							
Crude Model	Ref.	1.05 (0.68, 1.62)	0.62 (0.39, 0.98)	0.58 (0.37, 0.92)	0.77 (0.66, 0.91)	0.90 (0.84, 0.96)	0.004
Model 1	Ref.	0.99 (0.63, 1.55)	0.60 (0.38, 0.97)	0.53 (0.33, 0.86)	0.75 (0.64, 0.89)	0.89 (0.83, 0.96)	0.002
Model 2	Ref.	1.01 (0.64, 1.60)	0.65 (0.40, 1.05)	0.60 (0.36, 0.98)	0.78 (0.65, 0.93)	0.90 (0.84, 0.97)	0.014
Model 3	Ref.	1.00 (0.62, 1.62)	0.65 (0.39, 1.08)	0.57 (0.34, 0.96)	0.77 (0.64, 0.93)	0.90 (0.84, 0.97)	0.012

Model 1 adjusted for age, sex, education level, floating population, monthly income, and marital status; Model 2 further adjusted on the basis of Model 1 for BMI, smoking status, physical activity, and sedentary behavior; Model 3 further adjusted on the basis of Model 2 for duration of infection, CD4^+^ + T lymphocyte counts, complications, and medication side effects. SD, standard deviation; Q, quartiles; Ref., reference. Data are presented as odds ratios (95% confidence intervals).

**Figure 1:**
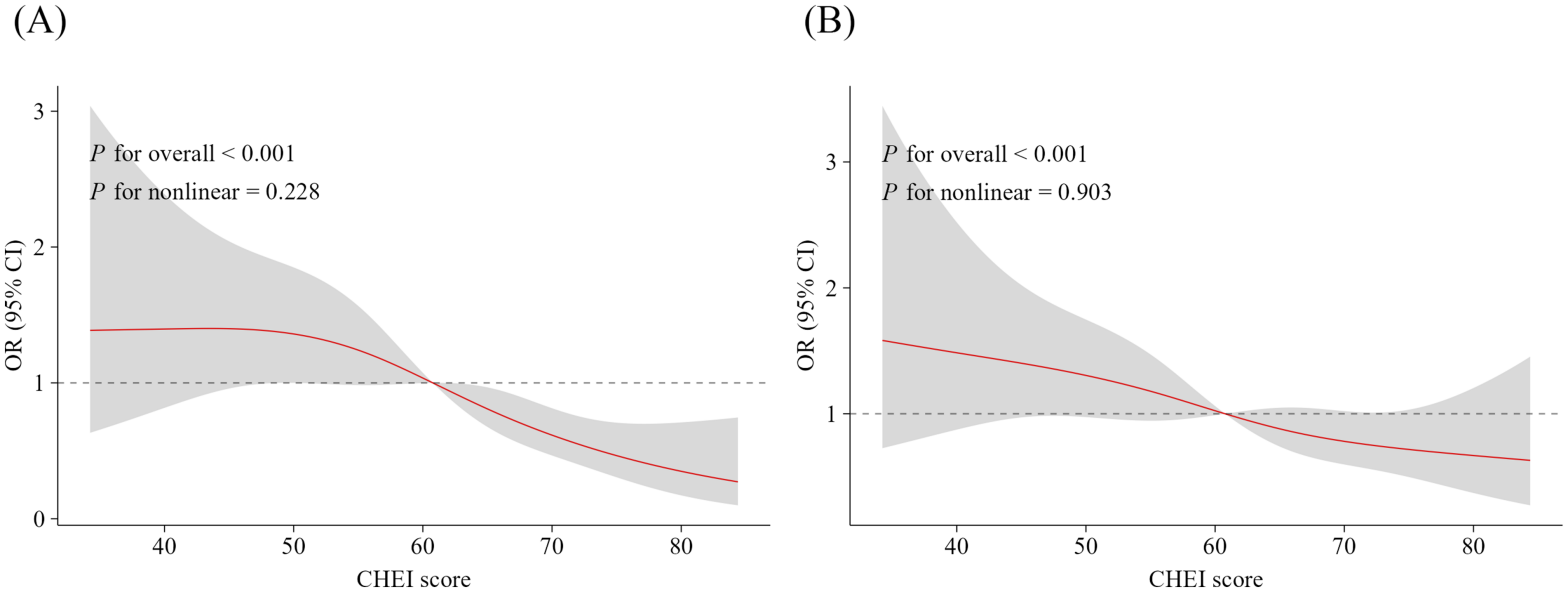
RCS analysis on the associations between total CHEI scores and the risk of experiencing depression **(A)** and anxiety **(B)** symptoms among people living with HIV. Adjusted for age, sex, education level, floating population, monthly income, marital status, body mass index, smoking status, physical activity, sedentary behavior, duration of infection, CD4^+^ T lymphocyte counts, complications, and medication side effects. The solid red line represents the multivariable-adjusted OR, and the gray area indicates the corresponding 95% confidential intervals. RCS, restricted cubic spline; CHEI, Chinese Healthy Eating Index.

The RCS analysis further indicated a relationship between total CHEI scores and the risk of depression (*p*_-non-linear_ = 0.228) and anxiety (*p*_-non-linear_ = 0.903) among PLHIV (Figure 1). As total CHEI scores exceeded 60, the risk of experiencing depression and anxiety symptoms showed a declining trend. Notably, the reduction in the risk of depression symptoms associated with higher total CHEI scores was significantly greater than that observed for anxiety symptoms.

### 3.3 Subgroup analyses

Further stratified analyses indicated that the protective association between total CHEI scores and the risk of depression symptoms remained consistent across various subgroups, including age, gender, education level, floating population status, marital status, overweight status, smoking status, physical activity, duration of infection, levels of CD4^+^ T lymphocyte counts, complications, and medication side effects (Table 3; *p*_-interaction_ ranged from 0.051 to 0.915). However, the association between total CHEI scores and depression risk was significantly stronger among individuals living with HIV with a monthly income of less than CNY ¥3,000 (*p*_-interaction_ = 0.030). Conversely, this association was weaker among PLHIV who exhibited sedentary behavior (*p* for interaction = 0.014). In stratified analyses examining the association between total CHEI scores and the risk of anxiety symptoms, no significant differences were observed across all subgroups (Table 4, *p*_-interaction_ ranged from 0.056 to 0.955).

**Table 3:** Association between total CHEI scores (per quartile increment) and risk of experiencing depression symptoms across various subgroups.

Subgroups	Total	Depression (n, %)	OR (95% CI)^a^	*p*_*-*interaction_ ^b^
Age				0.447
≥45 years	233	59 (25.32)	0.55 (0.38, 0.77)	
<45 years	467	141 (30.19)	0.77 (0.63, 0.95)	
Sex				0.761
Male	588	157 (26.7)	0.68 (0.56, 0.83)	
Female	112	43 (38.39)	0.53 (0.31, 0.85)	
Education level				0.422
Primary school or below	115	37 (32.17)	0.31 (0.14, 0.57)	
Junior middle school	168	53 (31.55)	0.58 (0.39, 0.84)	
Senior high school or vocational school	149	43 (28.86)	0.86 (0.53, 1.38)	
Junior college or above	268	67 (25.00)	0.83 (0.62, 1.10)	
Floating population status				0.676
No	341	94 (27.57)	0.68 (0.52, 0.89)	
Yes	359	106 (29.53)	0.71 (0.56, 0.90)	
Monthly income				0.030
≤CNY ¥ 3,000	141	58 (41.13)	0.36 (0.20, 0.58)	
CNY ¥ 3,001–5,000	216	54 (25.00)	0.78 (0.55, 1.09)	
CNY ¥5,001–8,000	209	49 (23.44)	0.66 (0.46, 0.91)	
> CNY ¥ 8,000	134	39 (29.10)	1.06 (0.67, 1.69)	
Marital status				0.854
Married	366	105 (28.69)	0.75 (0.59, 0.94)	
Unmarried	224	57 (25.45)	0.64 (0.45, 0.89)	
Divorce or widowhood	110	38 (34.55)	0.50 (0.26, 0.88)	
Overweight status				0.755
BMI < 24 kg/m^2^	476	142 (29.83)	0.68 (0.55, 0.83)	
BMI ≥ 24 kg/m^2^	224	58 (25.89)	0.73 (0.51, 1.03)	
Smoking status				0.260
No	498	133 (26.71)	0.66 (0.54, 0.82)	
Yes	202	67 (33.17)	0.82 (0.59, 1.13)	
Physical activity				0.308
Low level	417	134 (32.13)	0.74 (0.59, 0.92)	
Medium level	181	41 (22.65)	0.64 (0.42, 0.95)	
High level	102	25 (24.51)	0.52 (0.27, 0.91)	
Sedentary behavior				0.014
No	506	126 (24.9)	0.61 (0.49, 0.76)	
Yes	194	74 (38.14)	0.90 (0.65, 1.23)	
Duration of infection				0.805
≥6 years	360	97 (26.94)	0.74 (0.38, 0.77)	
<6 years	340	103 (30.29)	0.66 (0.50, 0.85)	
CD4^+^ T lymphocytes				0.051
<350/mm^3^	120	29 (24.17)	0.30 (0.11, 0.62)	
350–499/mm^3^	224	67 (29.91)	0.83 (0.62, 1.11)	
>500/mm^3^	326	96 (29.45)	0.70 (0.54, 0.90)	
Complications				0.911
No	497	137 (27.57)	0.71 (0.58, 0.87)	
Yes	203	63 (31.03)	0.65 (0.45, 0.93)	
Medication side effects				0.915
No	506	135 (26.68)	0.69 (0.55, 0.85)	
Yes	194	65 (33.51)	0.73 (0.52, 1.01)	

a Adjusted for age, sex, education level, floating population, monthly income, marital status, body mass index, smoking status, physical activity, sedentary behavior, infection duration, CD4^+^ T lymphocyte counts, complications, and medication side effects.

b The interaction was examined by adding an interaction term between the total CHEI scores and the stratification variable, as well as other risk factors.

BMI, body mass index. Data are presented as odds ratios (95% confidence intervals).

**Table 4:** Association between total average CHEI scores and risk of experiencing anxiety symptoms across various subgroups.

Subgroups	Total	Anxiety (n, %)	OR (95% CI)^a^	*p*_*-*interaction_ ^b^
Age				0.478
≥45 years	233	60 (25.8)	0.87 (0.63, 1.21)	
<45 years	467	160 (34.3)	0.80 (0.65, 0.98)	
Sex				0.642
Male	588	178 (30.3)	0.82 (0.69, 0.99)	
Female	112	42 (37.5)	0.69 (0.43, 1.09)	
Education level				0.774
Primary school or below	115	36 (31.3)	0.47 (0.25, 0.80)	
Junior middle school	168	53 (31.6)	0.83 (0.55, 1.22)	
Senior high school or vocational school	149	45 (30.2)	0.83 (0.52, 1.29)	
Junior college or above	268	86 (32.1)	0.81 (0.61, 1.06)	
Floating population				0.831
No	341	110 (32.3)	0.83 (0.65, 1.06)	
Yes	359	110 (30.6)	0.80 (0.63, 1.01)	
Monthly income				0.056
≤CNY ¥ 3,000	141	60 (42.6)	0.41 (0.22, 0.68)	
CNY ¥ 3,001–5,000	216	54 (25.0)	1.15 (0.81, 1.66)	
CNY ¥ 5,001–8,000	209	63 (30.1)	0.74 (0.53, 1.01)	
> CNY ¥ 8,000	134	43 (32.1)	0.83 (0.54, 1.27)	
Marital status				0.895
Married	366	122 (33.3)	0.83 (0.66, 1.04)	
Unmarried	224	67 (29.9)	0.76 (0.55, 1.05)	
Divorce or widowhood	110	31 (28.2)	0.89 (0.49, 1.61)	
Overweight status				0.467
BMI < 24 kg/m^2^	476	143 (30.0)	0.83 (0.67, 1.01)	
BMI ≥ 24 kg/m^2^	224	77 (34.4)	0.74 (0.53, 1.02)	
Smoking status				0.758
No	498	154 (30.9)	0.83 (0.68, 1.02)	
Yes	202	66 (32.7)	0.76 (0.54, 1.05)	
Physical activity				0.955
Low level	417	144 (34.5)	0.78 (0.63, 0.96)	
Medium level	181	45 (24.9)	0.90 (0.61, 1.31)	
High level	102	31 (30.4)	0.69 (0.38, 1.22)	
Sedentary behavior				0.818
No	506	139 (27.5)	0.78 (0.64, 0.96)	
Yes	194	81 (41.8)	0.79 (0.56, 1,11)	
Duration of infection				0.950
≥6 years	360	95 (26.4)	0.81 (0.64, 1.04)	
<6 years	340	125 (36.8)	0.79 (0.62, 1.00)	
CD4^+^ T lymphocytes				0.073
<350/mm^3^	120	39 (32.5)	0.51 (0.29, 0.83)	
350–499/mm^3^	224	71 (31.7)	0.88 (0.66, 1.18)	
>500/mm^3^	326	97 (29.8)	0.85 (0.65, 1.09)	
Complications				0.529
No	497	148 (29.8)	0.79 (0.65, 0.97)	
Yes	203	72 (35.5)	0.86 (0.62, 1.18)	
Medication side effects				0.198
No	506	147 (29.1)	0.85 (0.70, 1.04)	
Yes	194	73 (37.6)	0.67 (0.47, 0.93)	

a Adjusted for age, sex, education level, floating population, monthly income, marital status, body mass index, smoking status, physical activity, sedentary behavior, infection duration, CD4^+^ T lymphocyte counts, complications, and medication side effects.

b The interaction was examined by adding an interaction term between the total CHEI scores and the stratification variable, as well as other risk factors.

BMI, body mass index; Data are presented as odds ratios (95% confidence intervals).

### 3.4 Association of CHEI’S component scores with depression and anxiety symptoms

Supplementary Tables S1, S2 illustrate the associations between CHEI component scores and the risk of experiencing symptoms of depression and anxiety among PLHIV. After adjusting for potential confounding factors, higher CHEI component scores for dark vegetables (OR = 0.89; 95% CI: 0.80–1.00), fruits (OR = 0.89; 95% CI: 0.85–0.94) and dairy (OR = 0.88; 95% CI: 0.80–0.97), suggesting greater intake of these food groups, were associated with lower odds of experiencing depression symptoms. Conversely, higher scores indicating a lower intake of cooking oil were linked to a reduced likelihood of experiencing depression symptoms (OR = 0.94; 95% CI: 0.90–0.99).

Furthermore, increased consumption of total vegetables (OR = 0.89; 95% CI: 0.79–0.99), dark vegetables (OR = 0.86; 95% CI: 0.77–0.96), fruits (OR = 0.95; 95% CI: 0.90–0.99) and dairy (OR = 0.83; 95% CI: 0.76–0.91) was associated with lower odds of experiencing anxiety symptoms. Conversely, higher scores, indicating lower intake of alcohol, were linked to a decreased likelihood of experiencing anxiety symptoms (OR = 0.85; 95% CI: 0.730–0.99).

## 4 Discussion

Extensive research has demonstrated that an unhealthy dietary pattern may increase the risk of developing depression or anxiety, whereas a healthy dietary pattern may reduce this risk (28, 29). The present study reveals a significant inverse association between total CHEI scores and the likelihood of experiencing depression and anxiety symptoms among Chinese PLHIV. Specifically, an increase of one standard deviation (12 points) in the total CHEI score was associated with an approximately 33% reduction in the risk of experiencing depression symptoms and a 23% reduction in the risk of experiencing anxiety symptoms in this population. When total CHEI scores were more than 60, there was a notable decline in the risk of experiencing depression and anxiety symptoms. Subgroup analyses indicated that this association was strengthened among PLHIV who had a monthly income of less than CNY ¥ 3,000, whereas it was weakened among those with HIV who exhibited sedentary behavior. Furthermore, a higher intake of dark vegetables, fruits, and dairy was beneficial in reducing the risk of depression and anxiety symptoms in this population.

The CHEI is a measurement tool based on food entries and recommended intakes in the DGC, which was designed to assess long-term diet quality and evaluate adherence to the DGC (18, 30). To date, numerous studies have documented the impact of total CHEI scores on health outcomes and metabolic diseases (19–21, 31). A higher total CHEI score indicated better diet quality and greater adherence to dietary guidelines, reflecting sufficient consumption of whole grains, potatoes, vegetables, fruits, dairy products, and nuts, as well as lower intake of livestock and poultry meats, oils, salt, added sugar, and alcohol (32). According to the HEI-2015 classification, a score exceeding 70 was categorized as optimal adherence to the DGA-2015 (33). Our study was the first to evaluate diet quality among PLHIV using total CHEI scores. The total CHEI score for this population was 60.8, indicating moderate adherence to the DGC-2022 and relatively poor diet quality. The results largely aligned with the findings from studies conducted in other countries (34, 35), indicating that PLHIV tend to consume fewer vegetables, fruits, and dairy products. Dietary components appeared to be associated with the fecal microbiome and metabolome, which in turn influenced the health outcomes of PLWH (35). After adjusting for potential confounding factors, the results indicated that greater consumption of dark vegetables, fruits, and dairy products was associated with a reduced likelihood of experiencing symptoms of depression and anxiety among PLHIV. Additionally, a lower intake of cooking oil was linked to a decrease in the odds of experiencing depression symptoms, while reduced alcohol consumption was associated with a lower likelihood of experiencing anxiety symptoms. Consistent with the literature review, consumption of dark green vegetables, fruits, and dairy products was associated with protective effects against depression and anxiety (29, 36).

A growing body of evidence supports the notion that diet and nutrition significantly impact mental health and mental function throughout the lifespan (10). Although the determining factors of mental health are complex, emerging data indicate that high-quality diets are increasingly recognized as a promising strategy to address the growing prevalence of mood disorders, including depression and anxiety (10, 28, 29, 36). Our present study found that higher total CHEI scores, particularly elevated component scores for dark vegetables, fruits, and dairy products, were associated with a reduced likelihood of experiencing depression and anxiety symptoms among Chinese PLHIV. Notably, when total CHEI scores exceeded 60, there was a significant reduction in the risk of experiencing depression and anxiety symptoms. Furthermore, each one standard deviation increase of 12 points in the total CHEI score was linked to a significant decrease in the risk of depression and anxiety, further suggesting that a high-quality diet may be beneficial in mitigating the risk of mental health disorders in this population. Vegetables and fruits are rich sources of essential vitamins, antioxidant compounds, and dietary fiber, which may exert protective benefits against depression and anxiety by mitigating neuronal damage caused by oxidative stress and suppressing neuroinflammation processes (37, 38). Our previous report indicated that adherence to a vegetable-fruit pattern is associated with a reduced risk of anxiety and depression symptoms among PLHIV (39). Vegetables and fruits, along with their abundant bioactive compounds, may help alleviate chronic systemic inflammation and improve HIV-related immune dysregulation, thereby potentially decreasing the occurrence of mental health issues within this population. Dairy products, which contain abundant whey protein and calcium, have been shown to have an inverse association with depression (40). Whey protein is a rich source of tryptophan, which regulates mood behavior by increasing the contents of serotonin (41), and calcium plays a crucial role in excitatory transmission by moderating extracellular calcium fluctuations (42). Additionally, high intake of alcohol may increase the risk of experiencing anxiety-like behaviors by activating ferroptosis (43). Our findings provide support and additional evidence that higher diet quality assessed by CHEI was associated with a lower risk of experiencing depression and anxiety symptoms among PLHIV.

Interestingly, subgroup analyses indicated that the inverse association between total CHEI scores and depression risk was more pronounced among PLHIV with a monthly income of less than CNY ¥ 3,000, suggesting a potential effect modification based on income level. The present finding was consistent with previous evidence indicating that individuals with lower income were more likely to experience depression symptoms (44). One plausible explanation is that low-income individuals often face food insecurity and have limited access to affordable, high-quality food options (45). Under such resource-constrained conditions, improvements in diet quality may provide disproportionately greater benefits to mental wellbeing within this population. In contrast, the association between CHEI and depression symptoms was attenuated among PLHIV with sedentary behavior. This finding aligned with evidence suggesting that increased sitting time and sedentary behavior were associated with a heightened risk of depression (46, 47). This additionally suggests that sedentary behavior may significantly influence the relationship between total CHEI scores and depression.

The present study was the first to investigate the relationship between total CHEI scores and symptoms of depression and anxiety in PLHIV, thereby providing an opportunity for continuous dietary monitoring. However, several limitations should be considered when interpreting our findings. First, despite accounting for various potential confounding factors, our cross-sectional study design restricts the ability to establish causality between total CHEI scores and the risk of experiencing depression and anxiety symptoms among PLHIV. In addition, there may still be residual confounding from unmeasured factors, such as HIV-related stigma, social support, or specifics of ART regimens, all of which could potentially influence both diet and mental health outcomes. Therefore, future prospective cohort studies are necessary to provide evidence of this relationship while adjusting for as many confounding variables as possible. Second, the sample for the present study was obtained exclusively from a single city, Jinhua, using an accidental sampling method. This approach may restrict the representativeness of the sample; therefore, the generalizability of the findings should be interpreted with caution. Third, dietary intake was assessed using a semi-quantitative FFQ administered by trained medical graduate students. However, given the instrument’s moderate reliability (Cronbach’s *α* = 0.64) and potential recall bias, the interpretation of the findings should be approached with caution. Finally, depression and anxiety symptoms were evaluated using the HADS rather than through a clinician-administered structured diagnostic interview, which may result in potential overestimation or underestimation of the findings. Therefore, further investigation is needed to assess the applicability of our findings to PLHIV in other cities through a large-scale population study.

## 5 Implications for practice

Our findings underscore the significance of diet quality in the management of mental health of PLHIV, providing actionable insights for clinical and public health practices. It is suggested to incorporate routine nutritional screening into HIV clinical visits to identify nutritional issues early and implement interventions aimed at enhancing immune function and improving quality of life. Recognizing diet as a modifiable factor, healthcare providers can support PLHIV in improving mental health outcomes through dietary adjustments and interventions. Although longitudinal studies and intervention trials are essential for establishing causal relationships, our cross-sectional findings offer a compelling rationale for incorporating nutritional strategies into comprehensive HIV care.

## 6 Conclusion

The findings from the present study offer evidence indicating that both total CHEI scores and their component scores (specifically dark vegetables, fruits, and dairy products) were negatively associated with the likelihood of experiencing depression and anxiety symptoms among Chinese PLHIV. Specifically, when total CHEI scores exceeded 60, there was a significant reduction in the risk of experiencing these mental health symptoms. These findings suggest that high diet quality may be beneficial in reducing the risk of depression and anxiety symptoms among PLHIV. However, longitudinal studies are needed to confirm the causal relationship.

## Data Availability

The original contributions presented in the study are included in the article/Supplementary material, further inquiries can be directed to the corresponding authors.
