# Supplementary material for: Chinese healthy eating index as a measure of diet quality and its association with depression and anxiety symptoms among people living with HIV: a cross-sectional study in China
Source: Front Nutr. 2026 Feb 4;13:1748219. doi: 10.3389/fnut.2026.1748219 (PMC12913179; doi:10.3389/fnut.2026.1748219)
Supplement: Supplementary file 1 [file Data_Sheet_1.docx]

**Chinese Healthy Eating Index as a measure of diet quality and its association with depression and anxiety symptoms among people living with HIV: A cross-sectional study in China**

**Huiling Tang^1,†^, Zizheng Nie^2,†^, Chenyang Wu^2^, Junxia Cheng^1^, Ke Zhu^1^, Yingying Liu^2^, Mu Zhang^2^, Fengying Wang****^1*^ and Shufen Han^2*^**

^1^Jinhua Center for Disease Prevention and Control, Jinhua 321000, Zhejiang, P.R. China

^2^School of Public Health and Nursing, Hangzhou Normal University, Hangzhou 311121, Zhejiang, P.R. China

* Correspondence:
Corresponding Author: Fengying Wang; Shufen Han
Email: [wfy661015@163.com](mailto:wfy661015@163.com) (F. Wang); [sfhan@](mailto:sfhan@suda.edu.cn)hznu.edu.cn (S. Han)

**Table S1** Association of CHEI’s component scores with the risk of depression symptoms among people living with HIV

| **Components** | **Crude model** | |  | **Adjusted model** | |
| --- | --- | --- | --- | --- | --- |
|  | **OR (95%CI)** | ***P*** |  | **OR (95%CI)** | ***P*** |
| Total grain | 0.88 (0.73, 1.06) | 0.173 |  | 0.86 (0.70, 1.05) | 0.142 |
| Whole grains and mixed beans | 0.95 (0.86, 1.05) | 0.275 |  | 0.93 (0.84, 1.04) | 0.213 |
| Tubers | 0.94 (0.87, 1.03) | 0.193 |  | 0.95 (0.86, 1.04) | 0.291 |
| Total vegetables | 0.90 (0.81, 1.01) | 0.063 |  | 0.90 (0.80, 1.01) | 0.074 |
| Dark vegetables | **0.90 (0.81, 0.99)** | **0.033** |  | **0.89 (0.80, 1.00)** | **0.046** |
| Fruits | **0.91 (0.87, 0.96)** | **< 0.001** |  | **0.89 (0.85, 0.94)** | **< 0.001** |
| Dairy | **0.90 (0.83, 0.98)** | **0.014** |  | **0.88 (0.80, 0.97)** | **0.009** |
| Soybeans | 0.95 (0.87, 1.04) | 0.242 |  | 0.95 (0.86, 1.04) | 0.266 |
| Fish and seafood | 0.92 (0.84, 1.01) | 0.063 |  | 0.90 (0.82, 1.00) | 0.044 |
| Poultry | 0.96 (0.89, 1.04) | 0.352 |  | 0.96 (0.87, 1.05) | 0.358 |
| Eggs | 0.95 (0.86, 1.04) | 0.275 |  | 0.97 (0.87, 1.08) | 0.575 |
| Nuts | 0.96 (0.89, 1.04) | 0.315 |  | 0.98 (0.90, 1.07) | 0.675 |
| Red meat | 1.06 (0.97, 1.17) | 0.207 |  | 1.09 (0.98, 1.22) | 0.120 |
| Added sugars | 0.95 (0.83, 1.08) | 0.427 |  | 0.91 (0.79, 1.06) | 0.247 |
| Alcohol | 0.87 (0.76, 1.00) | 0.055 |  | 0.92 (0.79, 1.08) | 0.318 |
| Sodium | 1.00 (0.95, 1.06) | 0.897 |  | 1.02 (0.96, 1.08) | 0.533 |
| Cooking oil | **0.95 (0.91, 0.99)** | **0.012** |  | **0.94 (0.90, 0.99)** | **0.013** |

The adjusted model accounted for age, sex, education level, floating population, monthly income, marital status, body mass index, smoking, physical activity, sedentary behavior, infection duration, CD4^+^ T lymphocytes, complications and medication side effects.

**Table S2** Association of CHEI’s component scores with the risk of anxiety symptoms among people living with HIV

| **Components** | **Crude model** | |  | **Adjusted model** | |
| --- | --- | --- | --- | --- | --- |
|  | **OR (95%CI)** | ***P*** |  | **OR (95%CI)** | ***P*** |
| Total grain | 0.94 (0.78, 1.13) | 0.498 |  | 0.94 (0.77, 1.15) | 0.552 |
| Whole grains and mixed beans | 0.93 (0.85, 1.03) | 0.155 |  | 0.92 (0.83, 1.02) | 0.124 |
| Tubers | 1.03 (0.94, 1.11) | 0.561 |  | 1.03 (0.94, 1.13) | 0.472 |
| Total vegetables | **0.87 (0.79, 0.97)** | **0.012** |  | **0.89 (0.79, 0.99)** | **0.039** |
| Dark vegetables | **0.85 (0.77, 0.94)** | **0.001** |  | **0.86 (0.77, 0.96)** | **0.006** |
| Fruits | **0.95 (0.91, 0.99)** | **0.018** |  | **0.95 (0.90, 0.99)** | **0.029** |
| Dairy | **0.87 (0.80, 0.94)** | **< 0.001** |  | **0.83 (0.76, 0.91)** | **< 0.001** |
| Soybeans | 0.95 (0.87, 1.04) | 0.289 |  | 0.95 (0.87, 1.05) | 0.330 |
| Fish and seafood | 1.01 (0.93, 1.10) | 0.791 |  | 0.99 (0.90, 1.08) | 0.760 |
| Poultry | 1.05 (0.97, 1.13) | 0.229 |  | 1.03 (0.94, 1.13) | 0.494 |
| Eggs | 0.94 (0.86, 1.03) | 0.191 |  | 0.95 (0.86, 1.06) | 0.351 |
| Nuts | 0.94 (0.87, 1.02) | 0.128 |  | 0.96 (0.88, 1.04) | 0.303 |
| Red meat | 0.98 (0.90, 1.08) | 0.712 |  | 0.99 (0.89, 1.10) | 0.871 |
| Added sugars | 0.95 (0.83, 1.09) | 0.467 |  | 1.02 (0.88, 1.19) | 0.785 |
| Alcohol | **0.82 (0.72, 0.94)** | **0.005** |  | **0.85 (0.73, 0.99)** | **0.037** |
| Sodium | 1.02 (0.97, 1.07) | 0.529 |  | 1.03 (0.97, 1.09) | 0.317 |
| Cooking oil | 0.98 (0.94, 1.02) | 0.267 |  | 0.98 (0.93,1.03) | 0.360 |

The adjusted model accounted for age, sex, education level, floating population, monthly income, marital status, body mass index, smoking, physical activity, sedentary behavior, infection duration, CD4^+^ T lymphocytes, complications and medication side effects.
